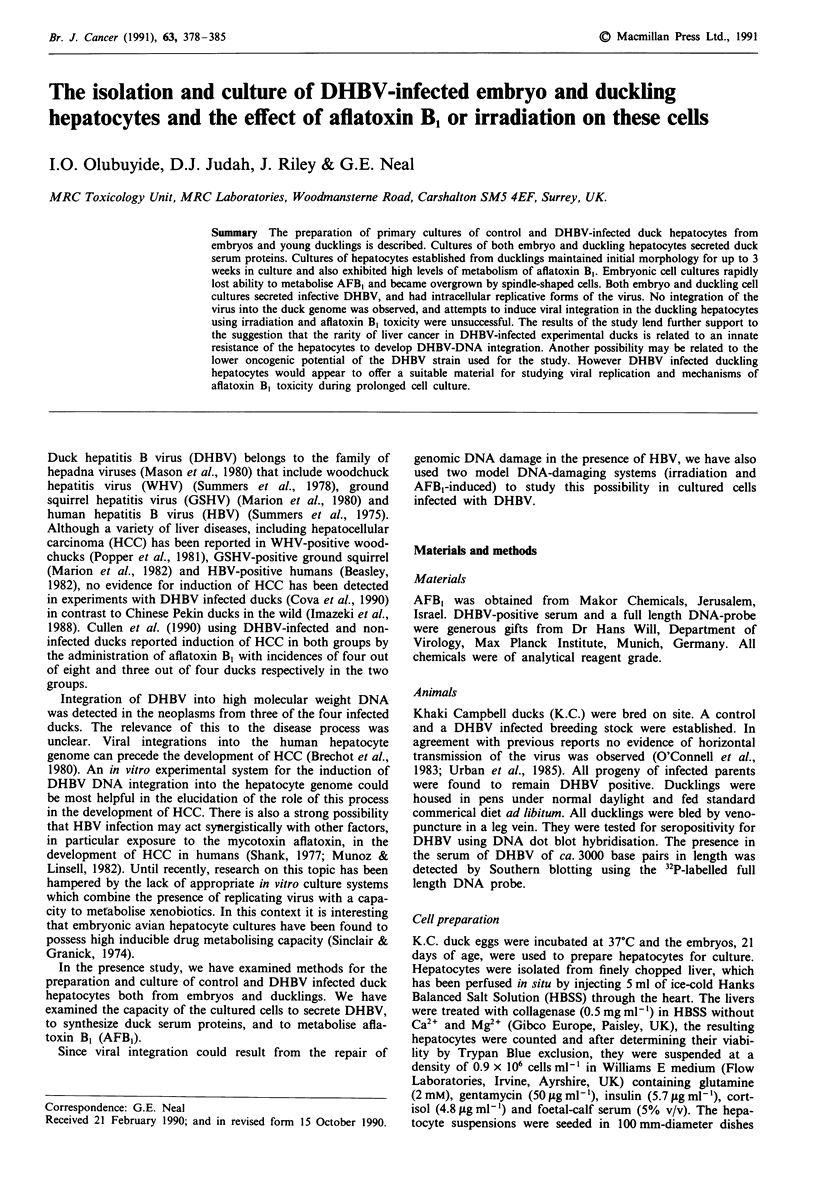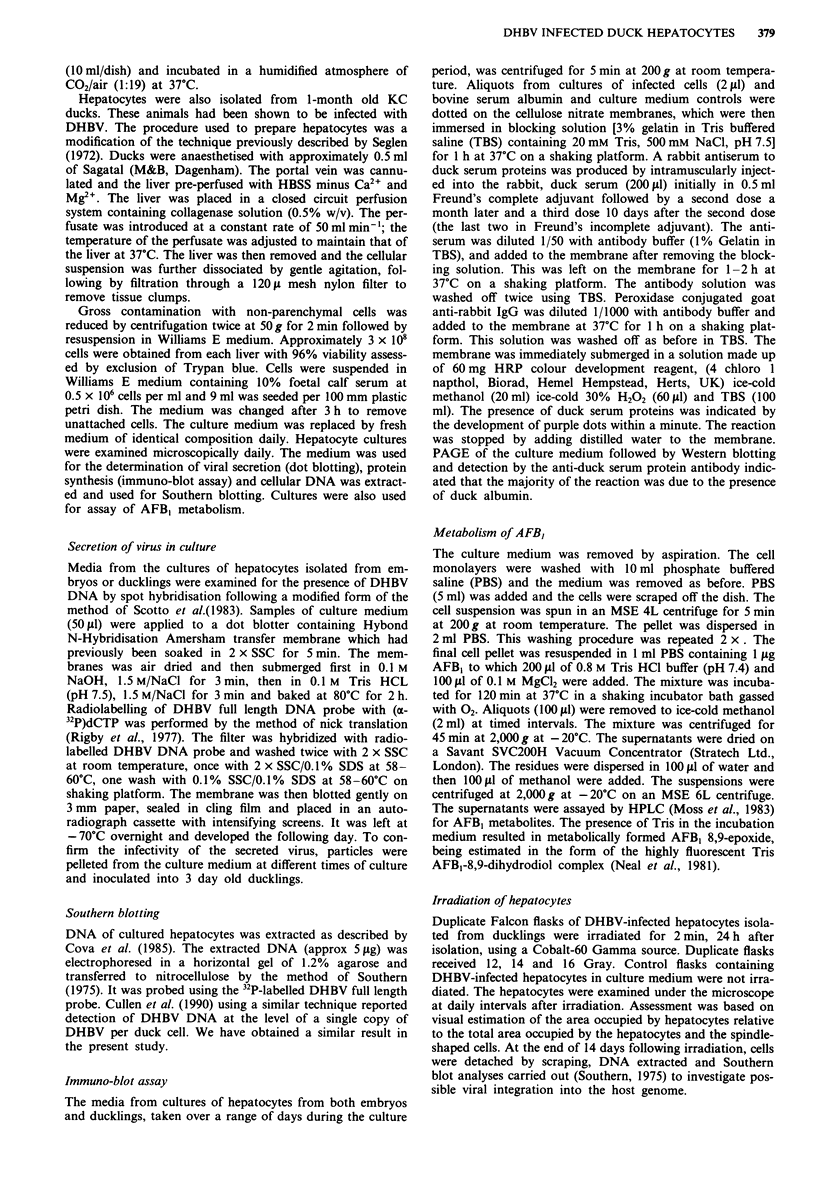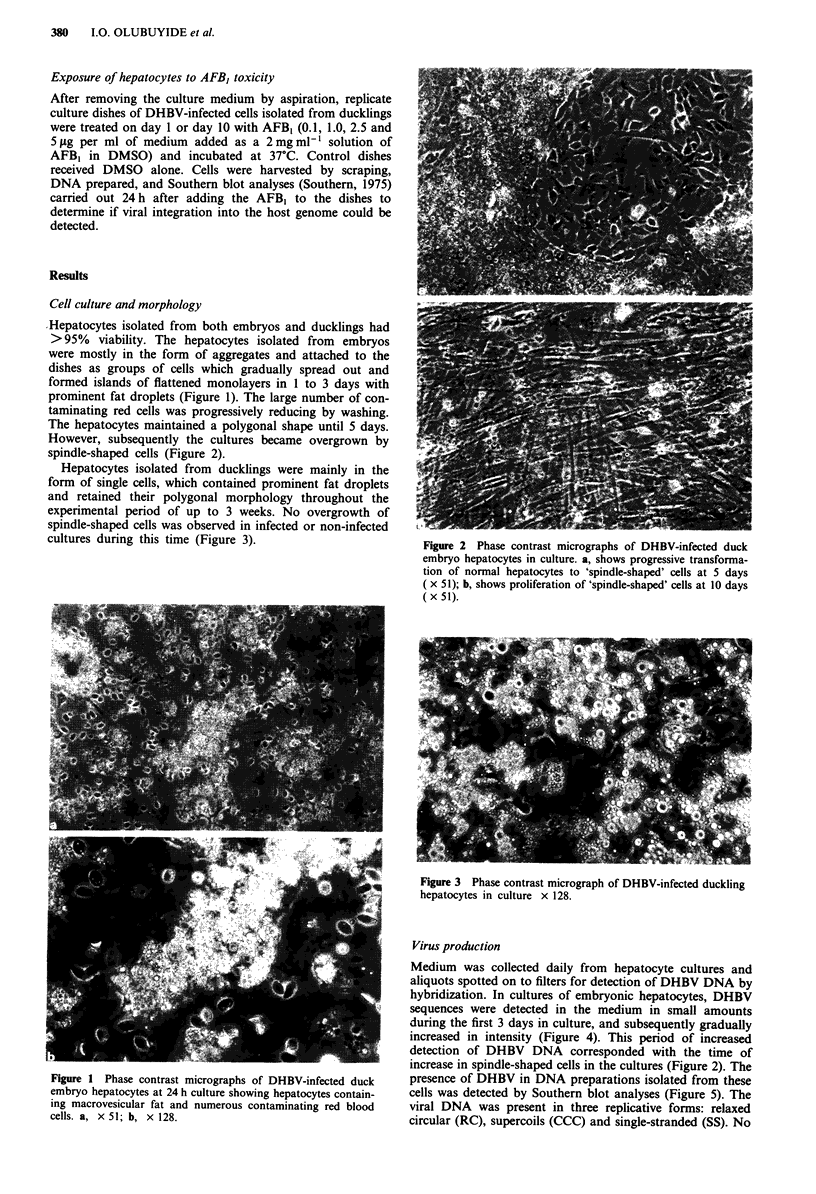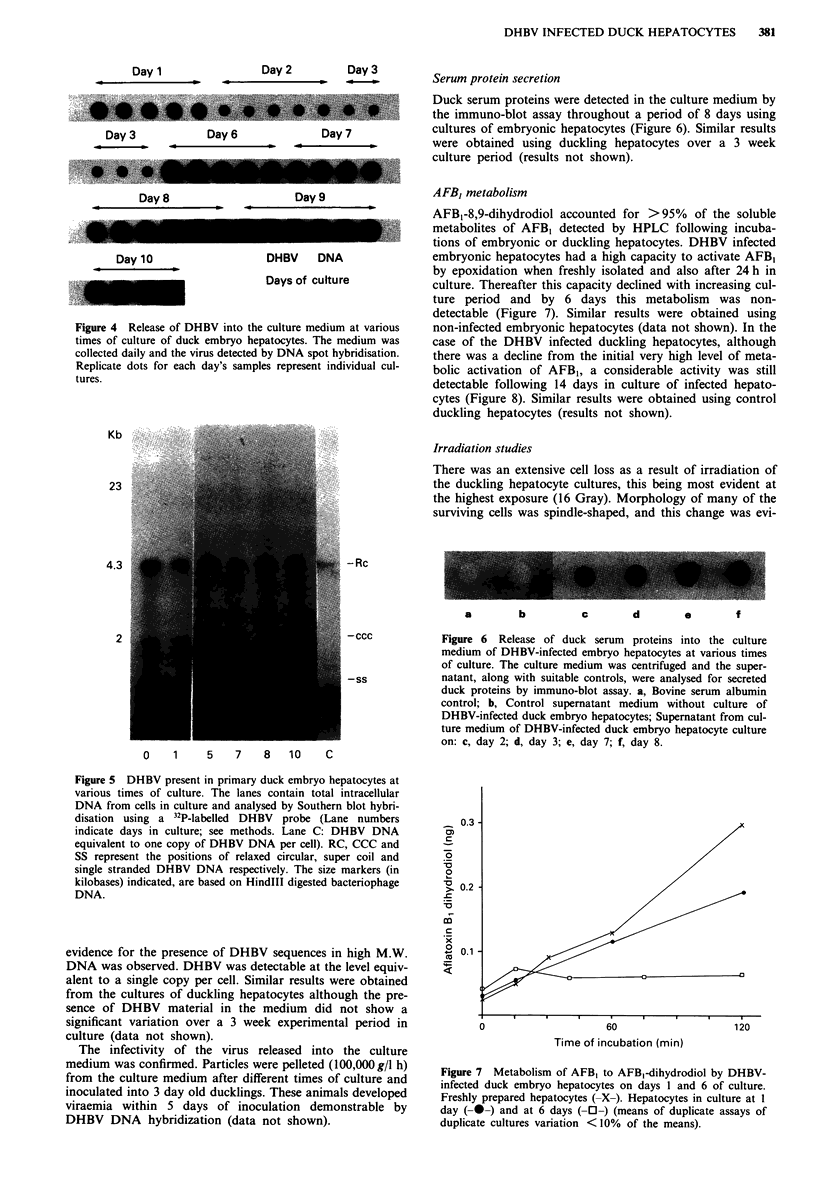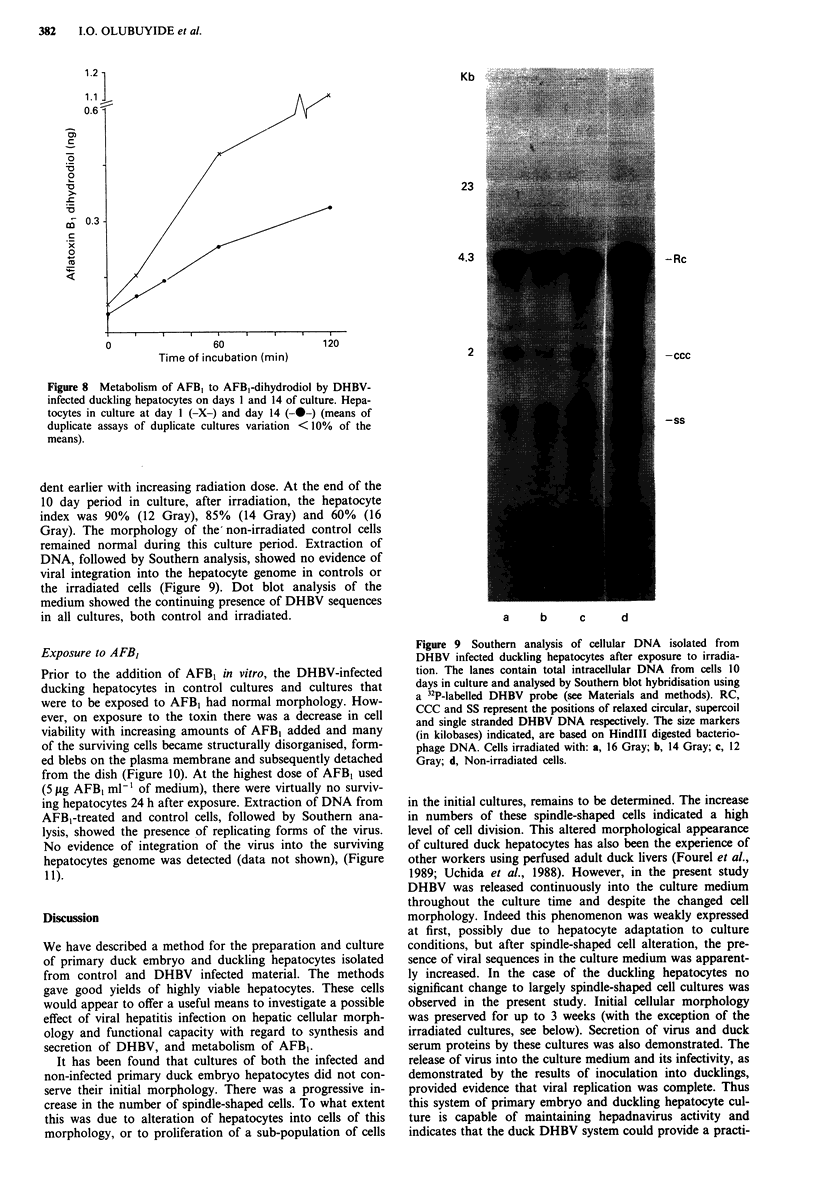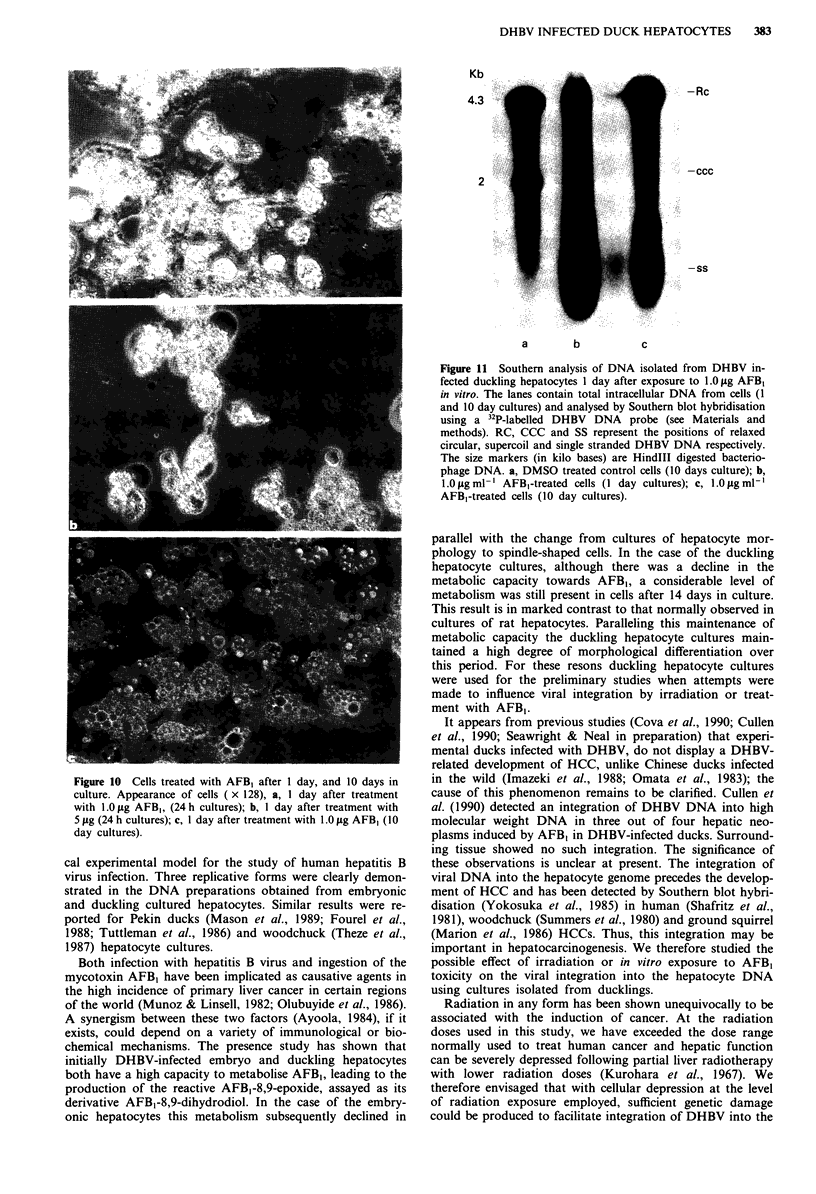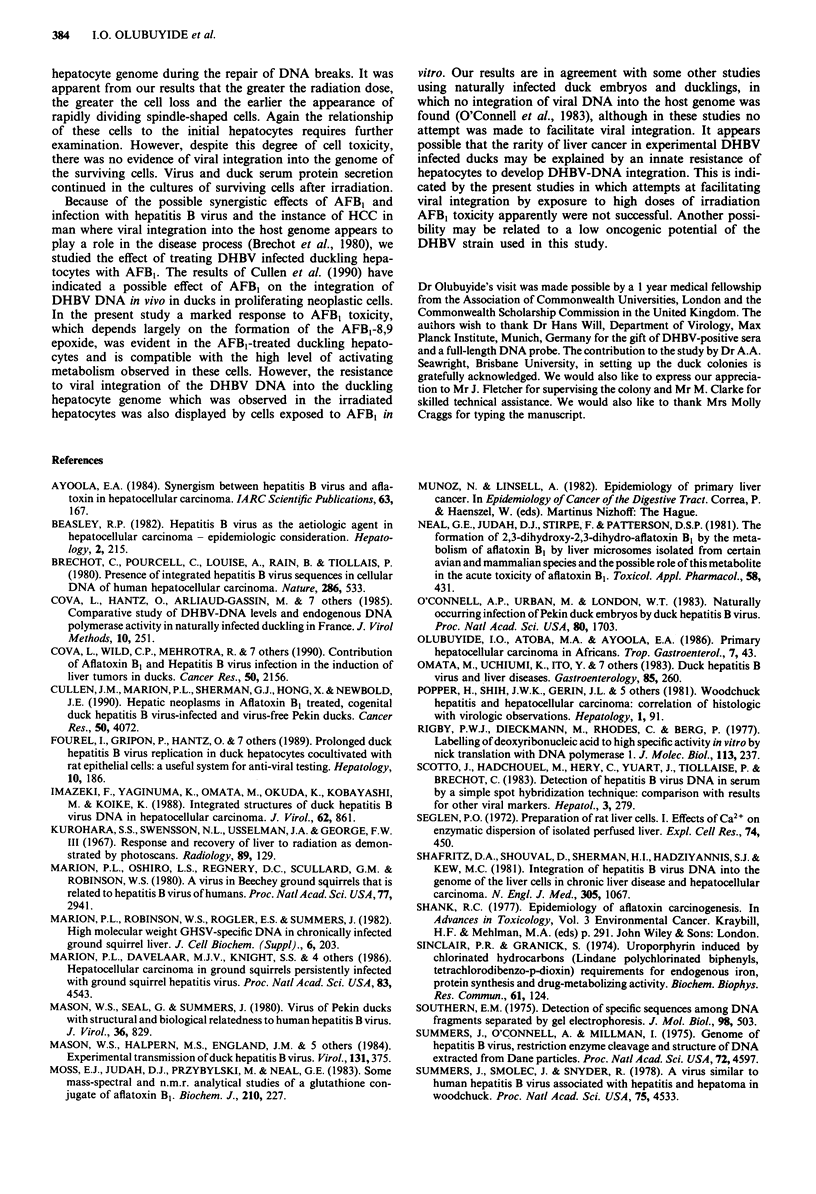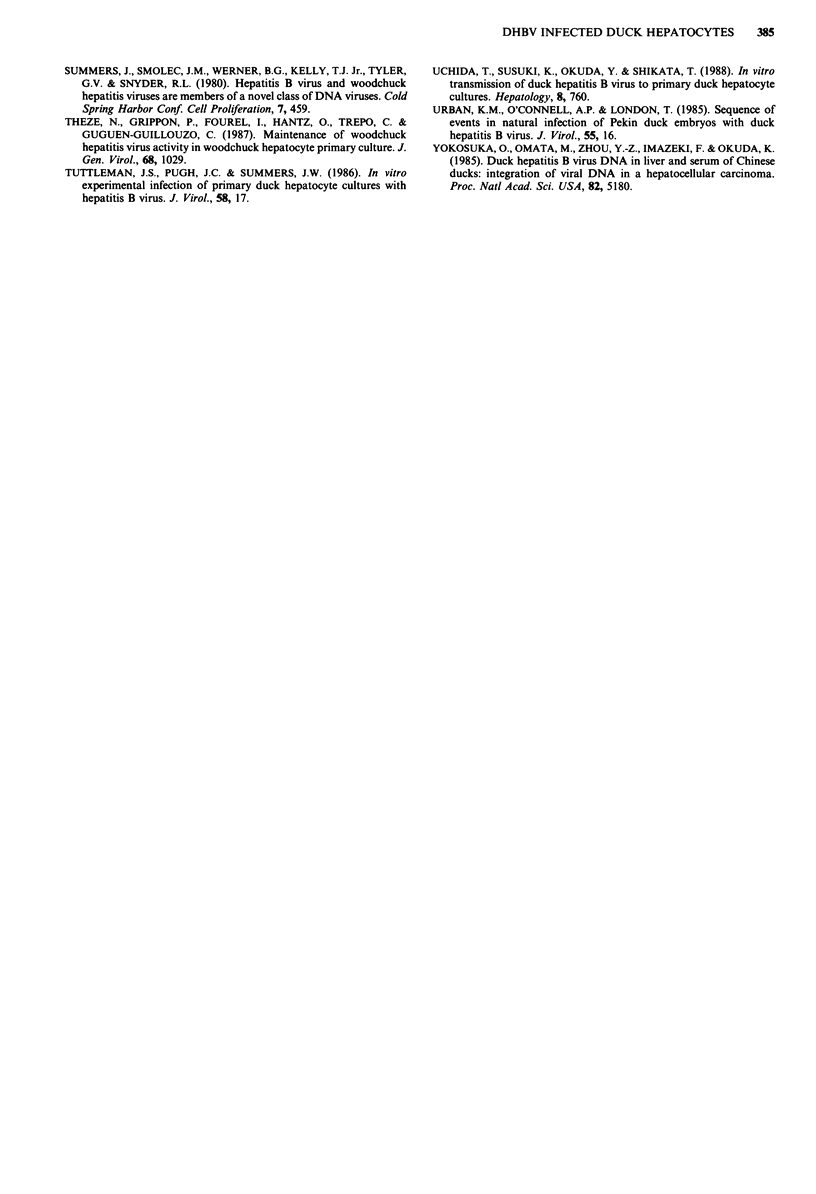# The isolation and culture of DHBV-infected embryo and duckling hepatocytes and the effect of aflatoxin B1 or irradiation on these cells.

**DOI:** 10.1038/bjc.1991.89

**Published:** 1991-03

**Authors:** I. O. Olubuyide, D. J. Judah, J. Riley, G. E. Neal

**Affiliations:** MRC Toxicology Unit, MRC Laboratories, Carshalton, Surrey, UK.

## Abstract

**Images:**


*Br. J. Cancer* (1991), **63**, 378–385 © Macmillan Press Ltd., 1991

### The isolation and culture of DHBV-infected embryo and duckling hepatocytes and the effect of aflatoxin $B_1$ or irradiation on these cells

I.O. Olubuyide, D.J. Judah, J. Riley & G.E. Neal

*MRC Toxicology Unit, MRC Laboratories, Woodmansterne Road, Carshalton SM5 4EF, Surrey, UK.*

**Summary** The preparation of primary cultures of control and DHBV-infected duck hepatocytes from embryos and young ducklings is described. Cultures of both embryo and duckling hepatocytes secreted duck serum proteins. Cultures of hepatocytes established from ducklings maintained initial morphology for up to 3 weeks in culture and also exhibited high levels of metabolism of aflatoxin $B_1$. Embryonic cell cultures rapidly lost ability to metabolise $AFB_1$ and became overgrown by spindle-shaped cells. Both embryo and duckling cell cultures secreted infective DHBV, and had intracellular replicative forms of the virus. No integration of the virus into the duck genome was observed, and attempts to induce viral integration in the duckling hepatocytes using irradiation and aflatoxin $B_1$ toxicity were unsuccessful. The results of the study lend further support to the suggestion that the rarity of liver cancer in DHBV-infected experimental ducks is related to an innate resistance of the hepatocytes to develop DHBV-DNA integration. Another possibility may be related to the lower oncogenic potential of the DHBV strain used for the study. However DHBV infected duckling hepatocytes would appear to offer a suitable material for studying viral replication and mechanisms of aflatoxin $B_1$ toxicity during prolonged cell culture.

Duck hepatitis B virus (DHBV) belongs to the family of hepadna viruses (Mason *et al.*, 1980) that include woodchuck hepatitis virus (WHV) (Summers *et al.*, 1978), ground squirrel hepatitis virus (GSHV) (Marion *et al.*, 1980) and human hepatitis B virus (HBV) (Summers *et al.*, 1975). Although a variety of liver diseases, including hepatocellular carcinoma (HCC) has been reported in WHV-positive woodchucks (Popper *et al.*, 1981), GSHV-positive ground squirrel (Marion *et al.*, 1982) and HBV-positive humans (Beasley, 1982), no evidence for induction of HCC has been detected in experiments with DHBV infected ducks (Cova *et al.*, 1990) in contrast to Chinese Pekin ducks in the wild (Imazeki *et al.*, 1988). Cullen *et al.* (1990) using DHBV-infected and non-infected ducks reported induction of HCC in both groups by the administration of aflatoxin $B_1$ with incidences of four out of eight and three out of four ducks respectively in the two groups.

Integration of DHBV into high molecular weight DNA was detected in the neoplasms from three of the four infected ducks. The relevance of this to the disease process was unclear. Viral integrations into the human hepatocyte genome can precede the development of HCC (Brechot *et al.*, 1980). An *in vitro* experimental system for the induction of DHBV DNA integration into the hepatocyte genome could be most helpful in the elucidation of the role of this process in the development of HCC. There is also a strong possibility that HBV infection may act synergistically with other factors, in particular exposure to the mycotoxin aflatoxin, in the development of HCC in humans (Shank, 1977; Munoz & Linsell, 1982). Until recently, research on this topic has been hampered by the lack of appropriate *in vitro* culture systems which combine the presence of replicating virus with a capacity to metabolise xenobiotics. In this context it is interesting that embryonic avian hepatocyte cultures have been found to possess high inducible drug metabolising capacity (Sinclair & Granick, 1974).

In the presence study, we have examined methods for the preparation and culture of control and DHBV infected duck hepatocytes both from embryos and ducklings. We have examined the capacity of the cultured cells to secrete DHBV, to synthesize duck serum proteins, and to metabolise aflatoxin $B_1$ ($AFB_1$).

Since viral integration could result from the repair of genomic DNA damage in the presence of HBV, we have also used two model DNA-damaging systems (irradiation and $AFB_1$-induced) to study this possibility in cultured cells infected with DHBV.

Correspondence: G.E. Neal
Received 21 February 1990; and in revised form 15 October 1990.

### Materials and methods

### Materials

$AFB_1$ was obtained from Makor Chemicals, Jerusalem, Israel. DHBV-positive serum and a full length DNA-probe were generous gifts from Dr Hans Will, Department of Virology, Max Planck Institute, Munich, Germany. All chemicals were of analytical reagent grade.

### Animals

Khaki Campbell ducks (K.C.) were bred on site. A control and a DHBV infected breeding stock were established. In agreement with previous reports no evidence of horizontal transmission of the virus was observed (O'Connell *et al.*, 1983; Urban *et al.*, 1985). All progeny of infected parents were found to remain DHBV positive. Ducklings were housed in pens under normal daylight and fed standard commerical diet *ad libitum*. All ducklings were bled by venopuncture in a leg vein. They were tested for seropositivity for DHBV using DNA dot blot hybridisation. The presence in the serum of DHBV of *ca.* 3000 base pairs in length was detected by Southern blotting using the $^{32}P$-labelled full length DNA probe.

### Cell preparation

K.C. duck eggs were incubated at 37°C and the embryos, 21 days of age, were used to prepare hepatocytes for culture. Hepatocytes were isolated from finely chopped liver, which has been perfused *in situ* by injecting 5 ml of ice-cold Hanks Balanced Salt Solution (HBSS) through the heart. The livers were treated with collagenase (0.5 mg ml$^{-1}$) in HBSS without $Ca^{2+}$ and $Mg^{2+}$ (Gibco Europe, Paisley, UK), the resulting hepatocytes were counted and after determining their viability by Trypan Blue exclusion, they were suspended at a density of $0.9 \times 10^6$ cells ml$^{-1}$ in Williams E medium (Flow Laboratories, Irvine, Ayrshire, UK) containing glutamine (2 mM), gentamycin (50 $\mu$g ml$^{-1}$), insulin (5.7 $\mu$g ml$^{-1}$), cortisol (4.8 $\mu$g ml$^{-1}$) and foetal-calf serum (5% v/v). The hepatocyte suspensions were seeded in 100 mm-diameter dishes

(10 ml/dish) and incubated in a humidified atmosphere of $CO_2$/air (1:19) at 37°C.

Hepatocytes were also isolated from 1-month old KC ducks. These animals had been shown to be infected with DHBV. The procedure used to prepare hepatocytes was a modification of the technique previously described by Seglen (1972). Ducks were anaesthetised with approximately 0.5 ml of Sagatal (M&B, Dagenham). The portal vein was cannulated and the liver pre-perfused with HBSS minus $Ca^{2+}$ and $Mg^{2+}$. The liver was placed in a closed circuit perfusion system containing collagenase solution (0.5% w/v). The perfusate was introduced at a constant rate of 50 ml $min^{-1}$; the temperature of the perfusate was adjusted to maintain that of the liver at 37°C. The liver was then removed and the cellular suspension was further dissociated by gentle agitation, following by filtration through a 120 $\mu$ mesh nylon filter to remove tissue clumps.

Gross contamination with non-parenchymal cells was reduced by centrifugation twice at 50 *g* for 2 min followed by resuspension in Williams E medium. Approximately $3 \times 10^8$ cells were obtained from each liver with 96% viability assessed by exclusion of Trypan blue. Cells were suspended in Williams E medium containing 10% foetal calf serum at $0.5 \times 10^6$ cells per ml and 9 ml was seeded per 100 mm plastic petri dish. The medium was changed after 3 h to remove unattached cells. The culture medium was replaced by fresh medium of identical composition daily. Hepatocyte cultures were examined microscopically daily. The medium was used for the determination of viral secretion (dot blotting), protein synthesis (immuno-blot assay) and cellular DNA was extracted and used for Southern blotting. Cultures were also used for assay of $AFB_1$ metabolism.

*Secretion of virus in culture*

Media from the cultures of hepatocytes isolated from embryos or ducklings were examined for the presence of DHBV DNA by spot hybridisation following a modified form of the method of Scotto *et al.*(1983). Samples of culture medium (50 $\mu$l) were applied to a dot blotter containing Hybond N-Hybridisation Amersham transfer membrane which had previously been soaked in 2 × SSC for 5 min. The membranes was air dried and then submerged first in 0.1 M NaOH, 1.5 M/NaCl for 3 min, then in 0.1 M Tris HCL (pH 7.5), 1.5 M/NaCl for 3 min and baked at 80°C for 2 h. Radiolabelling of DHBV full length DNA probe with ($\alpha$-$^{32}$P)dCTP was performed by the method of nick translation (Rigby *et al.*, 1977). The filter was hybridized with radiolabelled DHBV DNA probe and washed twice with 2 × SSC at room temperature, once with 2 × SSC/0.1% SDS at 58–60°C, one wash with 0.1% SSC/0.1% SDS at 58–60°C on shaking platform. The membrane was then blotted gently on 3 mm paper, sealed in cling film and placed in an autoradiograph cassette with intensifying screens. It was left at −70°C overnight and developed the following day. To confirm the infectivity of the secreted virus, particles were pelleted from the culture medium at different times of culture and inoculated into 3 day old ducklings.

*Southern blotting*

DNA of cultured hepatocytes was extracted as described by Cova *et al.* (1985). The extracted DNA (approx 5 $\mu$g) was electrophoresed in a horizontal gel of 1.2% agarose and transferred to nitrocellulose by the method of Southern (1975). It was probed using the $^{32}$P-labelled DHBV full length probe. Cullen *et al.* (1990) using a similar technique reported detection of DHBV DNA at the level of a single copy of DHBV per duck cell. We have obtained a similar result in the present study.

*Immuno-blot assay*

The media from cultures of hepatocytes from both embryos and ducklings, taken over a range of days during the culture period, was centrifuged for 5 min at 200 *g* at room temperature. Aliquots from cultures of infected cells (2 $\mu$l) and bovine serum albumin and culture medium controls were dotted on the cellulose nitrate membranes, which were then immersed in blocking solution [3% gelatin in Tris buffered saline (TBS) containing 20 mM Tris, 500 mM NaCl, pH 7.5] for 1 h at 37°C on a shaking platform. A rabbit antiserum to duck serum proteins was produced by intramuscularly injected into the rabbit, duck serum (200 $\mu$l) initially in 0.5 ml Freund's complete adjuvant followed by a second dose a month later and a third dose 10 days after the second dose (the last two in Freund's incomplete adjuvant). The antiserum was diluted 1/50 with antibody buffer (1% Gelatin in TBS), and added to the membrane after removing the blocking solution. This was left on the membrane for 1–2 h at 37°C on a shaking platform. The antibody solution was washed off twice using TBS. Peroxidase conjugated goat anti-rabbit IgG was diluted 1/1000 with antibody buffer and added to the membrane at 37°C for 1 h on a shaking platform. This solution was washed off as before in TBS. The membrane was immediately submerged in a solution made up of 60 mg HRP colour development reagent, (4 chloro 1 napthol, Biorad, Hemel Hempstead, Herts, UK) ice-cold methanol (20 ml) ice-cold 30% $H_2O_2$ (60 $\mu$l) and TBS (100 ml). The presence of duck serum proteins was indicated by the development of purple dots within a minute. The reaction was stopped by adding distilled water to the membrane. PAGE of the culture medium followed by Western blotting and detection by the anti-duck serum protein antibody indicated that the majority of the reaction was due to the presence of duck albumin.

*Metabolism of $AFB_1$*

The culture medium was removed by aspiration. The cell monolayers were washed with 10 ml phosphate buffered saline (PBS) and the medium was removed as before. PBS (5 ml) was added and the cells were scraped off the dish. The cell suspension was spun in an MSE 4L centrifuge for 5 min at 200 *g* at room temperature. The pellet was dispersed in 2 ml PBS. This washing procedure was repeated 2 ×. The final cell pellet was resuspended in 1 ml PBS containing 1 $\mu$g $AFB_1$ to which 200 $\mu$l of 0.8 M Tris HCl buffer (pH 7.4) and 100 $\mu$l of 0.1 M $MgCl_2$ were added. The mixture was incubated for 120 min at 37°C in a shaking incubator bath gassed with $O_2$. Aliquots (100 $\mu$l) were removed to ice-cold methanol (2 ml) at timed intervals. The mixture was centrifuged for 45 min at 2,000 *g* at −20°C. The supernatants were dried on a Savant SVC200H Vacuum Concentrator (Stratech Ltd., London). The residues were dispersed in 100 $\mu$l of water and then 100 $\mu$l of methanol were added. The suspensions were centrifuged at 2,000 *g* at −20°C on an MSE 6L centrifuge. The supernatants were assayed by HPLC (Moss *et al.*, 1983) for $AFB_1$ metabolites. The presence of Tris in the incubation medium resulted in metabolically formed $AFB_1$ 8,9-epoxide, being estimated in the form of the highly fluorescent Tris $AFB_1$-8,9-dihydrodiol complex (Neal *et al.*, 1981).

*Irradiation of hepatocytes*

Duplicate Falcon flasks of DHBV-infected hepatocytes isolated from ducklings were irradiated for 2 min, 24 h after isolation, using a Cobalt-60 Gamma source. Duplicate flasks received 12, 14 and 16 Gray. Control flasks containing DHBV-infected hepatocytes in culture medium were not irradiated. The hepatocytes were examined under the microscope at daily intervals after irradiation. Assessment was based on visual estimation of the area occupied by hepatocytes relative to the total area occupied by the hepatocytes and the spindle-shaped cells. At the end of 14 days following irradiation, cells were detached by scraping, DNA extracted and Southern blot analyses carried out (Southern, 1975) to investigate possible viral integration into the host genome.

*Exposure of hepatocytes to $AFB_1$ toxicity*

After removing the culture medium by aspiration, replicate culture dishes of DHBV-infected cells isolated from ducklings were treated on day 1 or day 10 with $AFB_1$ (0.1, 1.0, 2.5 and 5 μg per ml of medium added as a 2 mg $ml^{-1}$ solution of $AFB_1$ in DMSO) and incubated at 37°C. Control dishes received DMSO alone. Cells were harvested by scraping, DNA prepared, and Southern blot analyses (Southern, 1975) carried out 24 h after adding the $AFB_1$ to the dishes to determine if viral integration into the host genome could be detected.

### Results

*Cell culture and morphology*

Hepatocytes isolated from both embryos and ducklings had >95% viability. The hepatocytes isolated from embryos were mostly in the form of aggregates and attached to the dishes as groups of cells which gradually spread out and formed islands of flattened monolayers in 1 to 3 days with prominent fat droplets (Figure 1). The large number of contaminating red cells was progressively reducing by washing. The hepatocytes maintained a polygonal shape until 5 days. However, subsequently the cultures became overgrown by spindle-shaped cells (Figure 2).

Hepatocytes isolated from ducklings were mainly in the form of single cells, which contained prominent fat droplets and retained their polygonal morphology throughout the experimental period of up to 3 weeks. No overgrowth of spindle-shaped cells was observed in infected or non-infected cultures during this time (Figure 3).

**Figure 1** Phase contrast micrographs of DHBV-infected duck embryo hepatocytes at 24 h culture showing hepatocytes containing macrovesicular fat and numerous contaminating red blood cells. **a**, × 51; **b**, × 128.

**Figure 2** Phase contrast micrographs of DHBV-infected duck embryo hepatocytes in culture. **a**, shows progressive transformation of normal hepatocytes to 'spindle-shaped' cells at 5 days (× 51); **b**, shows proliferation of 'spindle-shaped' cells at 10 days (× 51).

**Figure 3** Phase contrast micrograph of DHBV-infected duckling hepatocytes in culture × 128.

*Virus production*

Medium was collected daily from hepatocyte cultures and aliquots spotted on to filters for detection of DHBV DNA by hybridization. In cultures of embryonic hepatocytes, DHBV sequences were detected in the medium in small amounts during the first 3 days in culture, and subsequently gradually increased in intensity (Figure 4). This period of increased detection of DHBV DNA corresponded with the time of increase in spindle-shaped cells in the cultures (Figure 2). The presence of DHBV in DNA preparations isolated from these cells was detected by Southern blot analyses (Figure 5). The viral DNA was present in three replicative forms: relaxed circular (RC), supercoils (CCC) and single-stranded (SS). No

**Figure 4** Release of DHBV into the culture medium at various times of culture of duck embryo hepatocytes. The medium was collected daily and the virus detected by DNA spot hybridisation. Replicate dots for each day's samples represent individual cultures.

**Figure 5** DHBV present in primary duck embryo hepatocytes at various times of culture. The lanes contain total intracellular DNA from cells in culture and analysed by Southern blot hybridisation using a $^{32}$P-labelled DHBV probe (Lane numbers indicate days in culture; see methods. Lane C: DHBV DNA equivalent to one copy of DHBV DNA per cell). RC, CCC and SS represent the positions of relaxed circular, super coil and single stranded DHBV DNA respectively. The size markers (in kilobases) indicated, are based on HindIII digested bacteriophage DNA.

evidence for the presence of DHBV sequences in high M.W. DNA was observed. DHBV was detectable at the level equivalent to a single copy per cell. Similar results were obtained from the cultures of duckling hepatocytes although the presence of DHBV material in the medium did not show a significant variation over a 3 week experimental period in culture (data not shown).

The infectivity of the virus released into the culture medium was confirmed. Particles were pelleted (100,000 *g*/1 h) from the culture medium after different times of culture and inoculated into 3 day old ducklings. These animals developed viraemia within 5 days of inoculation demonstrable by DHBV DNA hybridization (data not shown).

### *Serum protein secretion*

Duck serum proteins were detected in the culture medium by the immuno-blot assay throughout a period of 8 days using cultures of embryonic hepatocytes (Figure 6). Similar results were obtained using duckling hepatocytes over a 3 week culture period (results not shown).

### *$AFB_1$ metabolism*

$AFB_1$-8,9-dihydrodiol accounted for >95% of the soluble metabolites of $AFB_1$ detected by HPLC following incubations of embryonic or duckling hepatocytes. DHBV infected embryonic hepatocytes had a high capacity to activate $AFB_1$ by epoxidation when freshly isolated and also after 24 h in culture. Thereafter this capacity declined with increasing culture period and by 6 days this metabolism was non-detectable (Figure 7). Similar results were obtained using non-infected embryonic hepatocytes (data not shown). In the case of the DHBV infected duckling hepatocytes, although there was a decline from the initial very high level of metabolic activation of $AFB_1$, a considerable activity was still detectable following 14 days in culture of infected hepatocytes (Figure 8). Similar results were obtained using control duckling hepatocytes (results not shown).

### *Irradiation studies*

There was an extensive cell loss as a result of irradiation of the duckling hepatocyte cultures, this being most evident at the highest exposure (16 Gray). Morphology of many of the surviving cells was spindle-shaped, and this change was evi-

**Figure 6** Release of duck serum proteins into the culture medium of DHBV-infected embryo hepatocytes at various times of culture. The culture medium was centrifuged and the supernatant, along with suitable controls, were analysed for secreted duck proteins by immuno-blot assay. **a**, Bovine serum albumin control; **b**, Control supernatant medium without culture of DHBV-infected duck embryo hepatocytes; Supernatant from culture medium of DHBV-infected duck embryo hepatocyte culture on: **c**, day 2; **d**, day 3; **e**, day 7; **f**, day 8.

**Figure 7** Metabolism of $AFB_1$ to $AFB_1$-dihydrodiol by DHBV-infected duck embryo hepatocytes on days 1 and 6 of culture. Freshly prepared hepatocytes (-X-). Hepatocytes in culture at 1 day (-●-) and at 6 days (-□-) (means of duplicate assays of duplicate cultures variation <10% of the means).

**Figure 8** Metabolism of $AFB_1$ to $AFB_1$-dihydrodiol by DHBV-infected duckling hepatocytes on days 1 and 14 of culture. Hepatocytes in culture at day 1 (-X-) and day 14 (-●-) (means of duplicate assays of duplicate cultures variation $<10\%$ of the means).

dent earlier with increasing radiation dose. At the end of the 10 day period in culture, after irradiation, the hepatocyte index was 90% (12 Gray), 85% (14 Gray) and 60% (16 Gray). The morphology of the non-irradiated control cells remained normal during this culture period. Extraction of DNA, followed by Southern analysis, showed no evidence of viral integration into the hepatocyte genome in controls or the irradiated cells (Figure 9). Dot blot analysis of the medium showed the continuing presence of DHBV sequences in all cultures, both control and irradiated.

*Exposure to $AFB_1$*

Prior to the addition of $AFB_1$ *in vitro*, the DHBV-infected ducking hepatocytes in control cultures and cultures that were to be exposed to $AFB_1$ had normal morphology. However, on exposure to the toxin there was a decrease in cell viability with increasing amounts of $AFB_1$ added and many of the surviving cells became structurally disorganised, formed blebs on the plasma membrane and subsequently detached from the dish (Figure 10). At the highest dose of $AFB_1$ used (5 µg $AFB_1$ $ml^{-1}$ of medium), there were virtually no surviving hepatocytes 24 h after exposure. Extraction of DNA from $AFB_1$-treated and control cells, followed by Southern analysis, showed the presence of replicating forms of the virus. No evidence of integration of the virus into the surviving hepatocytes genome was detected (data not shown), (Figure 11).

### Discussion

We have described a method for the preparation and culture of primary duck embryo and duckling hepatocytes isolated from control and DHBV infected material. The methods gave good yields of highly viable hepatocytes. These cells would appear to offer a useful means to investigate a possible effect of viral hepatitis infection on hepatic cellular morphology and functional capacity with regard to synthesis and secretion of DHBV, and metabolism of $AFB_1$.

It has been found that cultures of both the infected and non-infected primary duck embryo hepatocytes did not conserve their initial morphology. There was a progressive increase in the number of spindle-shaped cells. To what extent this was due to alteration of hepatocytes into cells of this morphology, or to proliferation of a sub-population of cells

**Figure 9** Southern analysis of cellular DNA isolated from DHBV infected duckling hepatocytes after exposure to irradiation. The lanes contain total intracellular DNA from cells 10 days in culture and analysed by Southern blot hybridisation using a $^{32}P$-labelled DHBV probe (see Materials and methods). RC, CCC and SS represent the positions of relaxed circular, supercoil and single stranded DHBV DNA respectively. The size markers (in kilobases) indicated, are based on HindIII digested bacteriophage DNA. Cells irradiated with: **a**, 16 Gray; **b**, 14 Gray; **c**, 12 Gray; **d**, Non-irradiated cells.

in the initial cultures, remains to be determined. The increase in numbers of these spindle-shaped cells indicated a high level of cell division. This altered morphological appearance of cultured duck hepatocytes has also been the experience of other workers using perfused adult duck livers (Fourel *et al.*, 1989; Uchida *et al.*, 1988). However, in the present study DHBV was released continuously into the culture medium throughout the culture time and despite the changed cell morphology. Indeed this phenomenon was weakly expressed at first, possibly due to hepatocyte adaptation to culture conditions, but after spindle-shaped cell alteration, the presence of viral sequences in the culture medium was apparently increased. In the case of the duckling hepatocytes no significant change to largely spindle-shaped cell cultures was observed in the present study. Initial cellular morphology was preserved for up to 3 weeks (with the exception of the irradiated cultures, see below). Secretion of virus and duck serum proteins by these cultures was also demonstrated. The release of virus into the culture medium and its infectivity, as demonstrated by the results of inoculation into ducklings, provided evidence that viral replication was complete. Thus this system of primary embryo and duckling hepatocyte culture is capable of maintaining hepadnavirus activity and indicates that the duck DHBV system could provide a practi-

**Figure 10** Cells treated with $AFB_1$ after 1 day, and 10 days in culture. Appearance of cells ( × 128), **a**, 1 day after treatment with 1.0 μg $AFB_1$, (24 h cultures); **b**, 1 day after treatment with 5 μg (24 h cultures); **c**, 1 day after treatment with 1.0 μg $AFB_1$ (10 day cultures).

**Figure 11** Southern analysis of DNA isolated from DHBV infected duckling hepatocytes 1 day after exposure to 1.0 μg $AFB_1$ *in vitro*. The lanes contain total intracellular DNA from cells (1 and 10 day cultures) and analysed by Southern blot hybridisation using a $^{32}P$-labelled DHBV DNA probe (see Materials and methods). RC, CCC and SS represent the positions of relaxed circular, supercoil and single stranded DHBV DNA respectively. The size markers (in kilo bases) are HindIII digested bacteriophage DNA. **a**, DMSO treated control cells (10 days culture); **b**, 1.0 μg $ml^{-1}$ $AFB_1$-treated cells (1 day cultures); **c**, 1.0 μg $ml^{-1}$ $AFB_1$-treated cells (10 day cultures).

cal experimental model for the study of human hepatitis B virus infection. Three replicative forms were clearly demonstrated in the DNA preparations obtained from embryonic and duckling cultured hepatocytes. Similar results were reported for Pekin ducks (Mason *et al.*, 1989; Fourel *et al.*, 1988; Tuttleman *et al.*, 1986) and woodchuck (Theze *et al.*, 1987) hepatocyte cultures.

Both infection with hepatitis B virus and ingestion of the mycotoxin $AFB_1$ have been implicated as causative agents in the high incidence of primary liver cancer in certain regions of the world (Munoz & Linsell, 1982; Olubuyide *et al.*, 1986). A synergism between these two factors (Ayoola, 1984), if it exists, could depend on a variety of immunological or biochemical mechanisms. The presence study has shown that initially DHBV-infected embryo and duckling hepatocytes both have a high capacity to metabolise $AFB_1$, leading to the production of the reactive $AFB_1$-8,9-epoxide, assayed as its derivative $AFB_1$-8,9-dihydrodiol. In the case of the embryonic hepatocytes this metabolism subsequently declined in parallel with the change from cultures of hepatocyte morphology to spindle-shaped cells. In the case of the duckling hepatocyte cultures, although there was a decline in the metabolic capacity towards $AFB_1$, a considerable level of metabolism was still present in cells after 14 days in culture. This result is in marked contrast to that normally observed in cultures of rat hepatocytes. Paralleling this maintenance of metabolic capacity the duckling hepatocyte cultures maintained a high degree of morphological differentiation over this period. For these resons duckling hepatocyte cultures were used for the preliminary studies when attempts were made to influence viral integration by irradiation or treatment with $AFB_1$.

It appears from previous studies (Cova *et al.*, 1990; Cullen *et al.*, 1990; Seawright & Neal in preparation) that experimental ducks infected with DHBV, do not display a DHBV-related development of HCC, unlike Chinese ducks infected in the wild (Imazeki *et al.*, 1988; Omata *et al.*, 1983); the cause of this phenomenon remains to be clarified. Cullen *et al.* (1990) detected an integration of DHBV DNA into high molecular weight DNA in three out of four hepatic neoplasms induced by $AFB_1$ in DHBV-infected ducks. Surrounding tissue showed no such integration. The significance of these observations is unclear at present. The integration of viral DNA into the hepatocyte genome precedes the development of HCC and has been detected by Southern blot hybridisation (Yokosuka *et al.*, 1985) in human (Shafritz *et al.*, 1981), woodchuck (Summers *et al.*, 1980) and ground squirrel (Marion *et al.*, 1986) HCCs. Thus, this integration may be important in hepatocarcinogenesis. We therefore studied the possible effect of irradiation or *in vitro* exposure to $AFB_1$ toxicity on the viral integration into the hepatocyte DNA using cultures isolated from ducklings.

Radiation in any form has been shown unequivocally to be associated with the induction of cancer. At the radiation doses used in this study, we have exceeded the dose range normally used to treat human cancer and hepatic function can be severely depressed following partial liver radiotherapy with lower radiation doses (Kurohara *et al.*, 1967). We therefore envisaged that with cellular depression at the level of radiation exposure employed, sufficient genetic damage could be produced to facilitate integration of DHBV into the hepatocyte genome during the repair of DNA breaks. It was apparent from our results that the greater the radiation dose, the greater the cell loss and the earlier the appearance of rapidly dividing spindle-shaped cells. Again the relationship of these cells to the initial hepatocytes requires further examination. However, despite this degree of cell toxicity, there was no evidence of viral integration into the genome of the surviving cells. Virus and duck serum protein secretion continued in the cultures of surviving cells after irradiation.

Because of the possible synergistic effects of $AFB_1$ and infection with hepatitis B virus and the instance of HCC in man where viral integration into the host genome appears to play a role in the disease process (Brechot *et al.*, 1980), we studied the effect of treating DHBV infected duckling hepatocytes with $AFB_1$. The results of Cullen *et al.* (1990) have indicated a possible effect of $AFB_1$ on the integration of DHBV DNA *in vivo* in ducks in proliferating neoplastic cells. In the present study a marked response to $AFB_1$ toxicity, which depends largely on the formation of the $AFB_1$-8,9 epoxide, was evident in the $AFB_1$-treated duckling hepatocytes and is compatible with the high level of activating metabolism observed in these cells. However, the resistance to viral integration of the DHBV DNA into the duckling hepatocyte genome which was observed in the irradiated hepatocytes was also displayed by cells exposed to $AFB_1$ *in vitro*. Our results are in agreement with some other studies using naturally infected duck embryos and ducklings, in which no integration of viral DNA into the host genome was found (O'Connell *et al.*, 1983), although in these studies no attempt was made to facilitate viral integration. It appears possible that the rarity of liver cancer in experimental DHBV infected ducks may be explained by an innate resistance of hepatocytes to develop DHBV-DNA integration. This is indicated by the present studies in which attempts at facilitating viral integration by exposure to high doses of irradiation $AFB_1$ toxicity apparently were not successful. Another possibility may be related to a low oncogenic potential of the DHBV strain used in this study.

Dr Olubuyide's visit was made possible by a 1 year medical fellowship from the Association of Commonwealth Universities, London and the Commonwealth Scholarship Commission in the United Kingdom. The authors wish to thank Dr Hans Will, Department of Virology, Max Planck Institute, Munich, Germany for the gift of DHBV-positive sera and a full-length DNA probe. The contribution to the study by Dr A.A. Seawright, Brisbane University, in setting up the duck colonies is gratefully acknowledged. We would also like to express our appreciation to Mr J. Fletcher for supervising the colony and Mr M. Clarke for skilled technical assistance. We would also like to thank Mrs Molly Craggs for typing the manuscript.

### References

AYOOLA, E.A. (1984). Synergism between hepatitis B virus and aflatoxin in hepatocellular carcinoma. *IARC Scientific Publications*, **63**, 167.

BEASLEY, R.P. (1982). Hepatitis B virus as the aetiologic agent in hepatocellular carcinoma – epidemiologic consideration. *Hepatology*, **2**, 215.

BRECHOT, C., POURCELL, C., LOUISE, A., RAIN, B. & TIOLLAIS, P. (1980). Presence of integrated hepatitis B virus sequences in cellular DNA of human hepatocellular carcinoma. *Nature*, **286**, 533.

COVA, L., HANTZ, O., ARLIAUD-GASSIN, M. & 7 others (1985). Comparative study of DHBV-DNA levels and endogenous DNA polymerase activity in naturally infected duckling in France. *J. Virol Methods*, **10**, 251.

COVA, L., WILD, C.P., MEHROTRA, R. & 7 others (1990). Contribution of Aflatoxin $B_1$ and Hepatitis B virus infection in the induction of liver tumors in ducks. *Cancer Res.*, **50**, 2156.

CULLEN, J.M., MARION, P.L., SHERMAN, G.J., HONG, X. & NEWBOLD, J.E. (1990). Hepatic neoplasms in Aflatoxin $B_1$ treated, cogenital duck hepatitis B virus-infected and virus-free Pekin ducks. *Cancer Res.*, **50**, 4072.

FOUREL, I., GRIPON, P., HANTZ, O. & 7 others (1989). Prolonged duck hepatitis B virus replication in duck hepatocytes cocultivated with rat epithelial cells: a useful system for anti-viral testing. *Hepatology*, **10**, 186.

IMAZEKI, F., YAGINUMA, K., OMATA, M., OKUDA, K., KOBAYASHI, M. & KOIKE, K. (1988). Integrated structures of duck hepatitis B virus DNA in hepatocellular carcinoma. *J. Virol.*, **62**, 861.

KUROHARA, S.S., SWENSSON, N.L., USSELMAN, J.A. & GEORGE, F.W. III (1967). Response and recovery of liver to radiation as demonstrated by photoscans. *Radiology*, **89**, 129.

MARION, P.L., OSHIRO, L.S., REGNERY, D.C., SCULLARD, G.M. & ROBINSON, W.S. (1980). A virus in Beechey ground squirrels that is related to hepatitis B virus of humans. *Proc. Natl Acad. Sci. USA*, **77**, 2941.

MARION, P.L., ROBINSON, W.S., ROGLER, E.S. & SUMMERS, J. (1982). High molecular weight GHSV-specific DNA in chronically infected ground squirrel liver. *J. Cell Biochem. (Suppl)*., **6**, 203.

MARION, P.L., DAVELAAR, M.J.V., KNIGHT, S.S. & 4 others (1986). Hepatocellular carcinoma in ground squirrels persistently infected with ground squirrel hepatitis virus. *Proc. Natl Acad. Sci. USA*, **83**, 4543.

MASON, W.S., SEAL, G. & SUMMERS, J. (1980). Virus of Pekin ducks with structural and biological relatedness to human hepatitis B virus. *J. Virol.*, **36**, 829.

MASON, W.S., HALPERN, M.S., ENGLAND, J.M. & 5 others (1984). Experimental transmission of duck hepatitis B virus. *Virol.*, **131**, 375.

MOSS, E.J., JUDAH, D.J., PRZYBYLSKI, M. & NEAL, G.E. (1983). Some mass-spectral and n.m.r. analytical studies of a glutathione conjugate of aflatoxin $B_1$. *Biochem. J.*, **210**, 227.

MUNOZ, N. & LINSELL, A. (1982). Epidemiology of primary liver cancer. In *Epidemiology of Cancer of the Digestive Tract*. Correa, P. & Haenszel, W. (eds). Martinus Nizhoff: The Hague.

NEAL, G.E., JUDAH, D.J., STIRPE, F. & PATTERSON, D.S.P. (1981). The formation of 2,3-dihydroxy-2,3-dihydro-aflatoxin $B_1$ by the metabolism of aflatoxin $B_1$ by liver microsomes isolated from certain avian and mammalian species and the possible role of this metabolite in the acute toxicity of aflatoxin $B_1$. *Toxicol. Appl. Pharmacol.*, **58**, 431.

O'CONNELL, A.P., URBAN, M. & LONDON, W.T. (1983). Naturally occurring infection of Pekin duck embryos by duck hepatitis B virus. *Proc. Natl Acad. Sci. USA*, **80**, 1703.

OLUBUYIDE, I.O., ATOBA, M.A. & AYOOLA, E.A. (1986). Primary hepatocellular carcinoma in Africans. *Trop. Gastroenterol.*, **7**, 43.

OMATA, M., UCHIUMI, K., ITO, Y. & 7 others (1983). Duck hepatitis B virus and liver diseases. *Gastroenterology*, **85**, 260.

POPPER, H., SHIH, J.W.K., GERIN, J.L. & 5 others (1981). Woodchuck hepatitis and hepatocellular carcinoma: correlation of histologic with virologic observations. *Hepatology*, **1**, 91.

RIGBY, P.W.J., DIECKMANN, M., RHODES, C. & BERG, P. (1977). Labelling of deoxyribonucleic acid to high specific activity *in vitro* by nick translation with DNA polymerase 1. *J. Molec. Biol.*, **113**, 237.

SCOTTO, J., HADCHOUEL, M., HERY, C., YUART, J., TIOLLAISE, P. & BRECHOT, C. (1983). Detection of hepatitis B virus DNA in serum by a simple spot hybridization technique: comparison with results for other viral markers. *Hepatol.*, **3**, 279.

SEGLEN, P.O. (1972). Preparation of rat liver cells. I. Effects of $Ca^{2+}$ on enzymatic dispersion of isolated perfused liver. *Expl. Cell Res.*, **74**, 450.

SHAFRITZ, D.A., SHOUVAL, D., SHERMAN, H.I., HADZIYANNIS, S.J. & KEW, M.C. (1981). Integration of hepatitis B virus DNA into the genome of the liver cells in chronic liver disease and hepatocellular carcinoma. *N. Engl. J. Med.*, **305**, 1067.

SHANK, R.C. (1977). Epidemiology of aflatoxin carcinogenesis. In *Advances in Toxicology*, Vol. 3 Environmental Cancer. Kraybill, H.F. & Mehlman, M.A. (eds) p. 291. John Wiley & Sons: London.

SINCLAIR, P.R. & GRANICK, S. (1974). Uroporphyrin induced by chlorinated hydrocarbons (Lindane polychlorinated biphenyls, tetrachlorodibenzo-p-dioxin) requirements for endogenous iron, protein synthesis and drug-metabolizing activity. *Biochem. Biophys. Res. Commun.*, **61**, 124.

SOUTHERN, E.M. (1975). Detection of specific sequences among DNA fragments separated by gel electrophoresis. *J. Mol. Biol.*, **98**, 503.

SUMMERS, J., O'CONNELL, A. & MILLMAN, I. (1975). Genome of hepatitis B virus, restriction enzyme cleavage and structure of DNA extracted from Dane particles. *Proc. Natl Acad. Sci. USA*, **72**, 4597.

SUMMERS, J., SMOLEC, J. & SNYDER, R. (1978). A virus similar to human hepatitis B virus associated with hepatitis and hepatoma in woodchuck. *Proc. Natl Acad. Sci. USA*, **75**, 4533.

SUMMERS, J., SMOLEC, J.M., WERNER, B.G., KELLY, T.J. Jr., TYLER, G.V. & SNYDER, R.L. (1980). Hepatitis B virus and woodchuck hepatitis viruses are members of a novel class of DNA viruses. *Cold Spring Harbor Conf. Cell Proliferation*, **7**, 459.

THEZE, N., GRIPPON, P., FOUREL, I., HANTZ, O., TREPO, C. & GUGUEN-GUILLOUZO, C. (1987). Maintenance of woodchuck hepatitis virus activity in woodchuck hepatocyte primary culture. *J. Gen. Virol.*, **68**, 1029.

TUTTLEMAN, J.S., PUGH, J.C. & SUMMERS, J.W. (1986). *In vitro* experimental infection of primary duck hepatocyte cultures with hepatitis B virus. *J. Virol.*, **58**, 17.

UCHIDA, T., SUSUKI, K., OKUDA, Y. & SHIKATA, T. (1988). *In vitro* transmission of duck hepatitis B virus to primary duck hepatocyte cultures. *Hepatology*, **8**, 760.

URBAN, K.M., O'CONNELL, A.P. & LONDON, T. (1985). Sequence of events in natural infection of Pekin duck embryos with duck hepatitis B virus. *J. Virol.*, **55**, 16.

YOKOSUKA, O., OMATA, M., ZHOU, Y.-Z., IMAZEKI, F. & OKUDA, K. (1985). Duck hepatitis B virus DNA in liver and serum of Chinese ducks: integration of viral DNA in a hepatocellular carcinoma. *Proc. Natl Acad. Sci. USA*, **82**, 5180.